# DESCARTES and his project of a fantasized brain

**DOI:** 10.1590/1980-57642021dn15-020017

**Published:** 2021

**Authors:** Eliasz Engelhardt

**Affiliations:** 1 Institute of Neurology Deolindo Couto, Universidade Federal do Rio de Janeiro - Rio de Janeiro, RJ, Brazil.; 2 Institute of Psychiatry, Universidade Federal do Rio de Janeiro - Rio de Janeiro, RJ, Brazil.

**Keywords:** Descartes, brain, pineal gland, animal spirits, microscopy, Descartes, cérebro, glândula pineal, espíritos animais, microscopia

## Abstract

Interest in anatomy dates from the earliest times. Such knowledge was acquired through dissections of animals and human corpses by many researchers. The macroscopic anatomy of the varied structures of the brain were identified over the centuries, and the predominating solid substance was seen as amorphous, and devoid of any specific function, until the Renaissance. René Descartes, a personage with a brilliant and creative mind, conceived the brain, its structure and function, in a distinct manner to what was known at his time. He valued the solid matter and gave it, for the first time, a theoretical minute structure, related to a presumptive function based on the presence of the pineal gland and the animal spirits, underlying cognitive, sensory and motor activities. Such structural view was endorsed, in a given sense, by the microscopic findings of Marcello Malpighi, which begun to change the understanding of the nervous system.

## RESUMO.

O interesse pela anatomia data desde a mais remota antiguidade. Esse conhecimento foi adquirido por meio de dissecções em animais e cadáveres humanos, por muitos pesquisadores. A anatomia macroscópica de variadas estruturas do cérebro foi identificada com o passar dos séculos, e a substância sólida predominante foi vista como amorfa e destituída de qualquer função específica até o período do Renascimento. René Descartes, um personagem com uma mente brilhante e criativa, concebeu o cérebro, sua estrutura e função, de um modo distinto ao que era conhecido no seu tempo. Ele valorizou e deu à substância sólida, pela primeira vez, uma estrutura teórica formada por elementos minúsculos, relacionada a uma função presuntiva, baseada na presença da glândula pineal e dos espíritos animais, subjacentes às atividades cognitiva, sensorial e motora. Essa vista estrutural foi, de certa maneira, endossada pelos achados microscópicos de Marcello Malpighi, o que começou a modificar a compreensão do sistema nervoso.

## INTRODUCTION

Interest in anatomy dates from the most ancient times. Such knowledge was acquired through dissections of animals and human corpses by many researchers. Among those that must be cited are the pioneer studies of Aristotle (4^th^ century BC) on animals, and Herophilus and Erasistratus (4^th^-3^rd^ centuries BC) on human corpses. They were followed, much later, by two of the most relevant personalities in the history of anatomy, Claudius Galenus (2^nd^-3^rd^ century AD), who established solid anatomic knowledge based on animal dissections, which lasted for more than a millennium, and Andreas Vesalius (16^th^ century), with his milestone work on human anatomy.^1^ ^,^ ^2^ ^,^ ^3^ The anatomical findings were frequently accompanied by functional conjectures, and after a cardiocentric vs encephalocentric quest, where the latter prevailed, the ventricular system was chosen to house the faculties of the soul, as clearly postulated by Nemesius of Emesa (4^th^ century AD), with his “ventricular doctrine”. Such view lasted for the entire Middle Ages and also during the Renaissance, and it was maintained even when the first human dissections reappeared, with Mondinus de Liuzzi (15^th^ century).^4^

**Figure 1.**
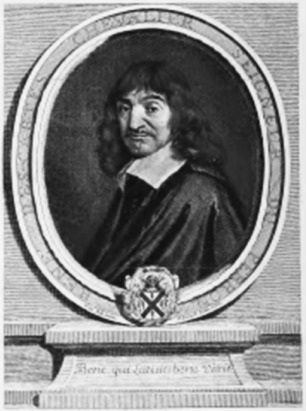
Portrait of René Descartes by Frans Hals.^6^

Those were the anatomical resources and functional propositions, when René Descartes (1596-1650), French math ematician, physicist, and philosopher, made his scientific appearance (Figure 1).^5^ ^,^ ^6^

Descartes intended to give a physical theory of the universe to explain all the phenomena of nature, and with that purpose, he produced a text, between 1629 and 1633, planned to be published as a book (*Le Monde*), where one part should be about man (*L’Homme*).^7^ ^,^ ^8^ The manuscript was almost finished, but in view of the condemnation of Galileo Galilei for religious reasons due to his “heretical” writings (1633), and afraid of similar consequences, he gave up the plan of publishing this text.^7^ ^,^ ^8^ A time later, he ceded to his scientific nature and resumed the work with the help of Gerard van Gutschoven as prosector and personal assistant. He performed a detailed study on the anatomy of the brain, initially of animals (especially sheep), and later also of humans, after which he made a revision of the manuscript (1640).^7^ ^,^ ^8^ ^,^ ^9^ Apparently, unsatisfied with this state of affairs, he published parts of the text he considered as “safe” in his *Discourse* (1637), *La Dioptrique* (1637), and *Les Passions de L’Âme* (1649). However, he retained most of the original manuscript in his possession until his death.^5^ ^,^ ^7^ ^,^ ^9^ ^,^ ^10^ ^,^ ^11^

The unpublished manuscript of *L’Homme* left by Descartes was written in French, and the possibly existing figures were not found. Translated to Latin by the physician Florentius Schuyl, it was published posthumously (1662). A version in French was published (1664) by his “literary executor” Claude Creselier, with remarks by the physician Louis de La Forge along with inclusion of figures produced by the latter and his former assistant van Gutschoven, who became a physician, anatomist, and mathematician, already acquainted with this work, reflecting the text as faithfully as possible.^12^ ^,^ ^13^ ^,^ ^14^ ^,^ ^15^ ^,^ ^16^

Here, some aspects of Descartes’ peculiar view on the brain, its structure and function, are appreciated.

## THE STRUCTURE OF THE CARTESIAN BRAIN

The published anatomical works at the time, it must be stressed, did not offer a characterization, except for the macroscopic features, of the structure of the substance of the brain, which remained an amorphous (unshaped) matter. A distinct view was presented by Descartes. Although he was not an anatomist, he committed a good time to consult anatomical studies (by Vesalius and others), watching butchers work, and personally dissecting various kinds of animals, and also human material, according to letters to his friend Marin Mersenne (1632, 1939).^4^ ^,^ ^6^ ^,^ ^7^ ^,^ ^9^

Descartes writes about an imaginary man, a conceptual model, stating that: “These men will be composed, as we are, of a soul and a body... I will show you how these two natures must be joined and united so as to compose men who resemble us…”. And then: “…I suppose that the body is nothing more than a statue or a machine made of earth…”, and compared it to other similar machines such as clocks, fountains, mills. Regarding the soul, he wrote: “…when the rational soul will be united to such machine, it will receive a key place inside the brain…”.^16^

**Figure 2.**
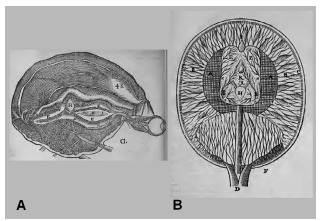
Figures of the brain illustrating Descartes’ description.^16^ ^,^ ^17^ (A) Drawing featuring a tridimensional brain, based probably on a sheep brain (provided by van Gutschoven [G]). (B) Diagrammatic schema featuring a horizontal view of the brain (provided by La Forge [F]).

He describes the brain, in a hypothetical manner, as constituted by a substance forming broad walls, the solid part of the brain [medulla of the brain, according to La Forge], defined as “a tissue composed in a certain particular way” [nervous tissue], surrounding the cavities [*concauitez du cerveau*] [ventricles] (EE) [conceived as a single continuous cavity, according to La Forge]. The internal part of the solid matter (AA), in direct contact with the ventricles, is formed by filaments (“small tubules”) (*petits tuyaux*) that constitute a thick and compact net. Many very delicate filaments of unequal lengths originate from this net, where some occupy an external space (BB), interweaved in various ways, leaving between them intervals or “pores”; others course to the peripheral space (surface) (CC), each ending in the extremity of small vessels that are there, and the longest converge from each side to form a stalk-like structure (D) (Figures 2A and 2B).^16^ ^,^ ^17^ The middle of the brain is occupied by the gland H [pineal gland]. The whole structure is enveloped by a double membrane [pia and dura mater, according to La Forge]. The stalk, enclosed by an extension of the double membrane is followed by a longer projection [spinal cord], from which emerge nerves destined for the trunk and the limbs.^16^

H: pineal gland; AA: inner solid part; BB: outer solid part; EE: cavities; D: stem-like structure; aa: small tubules; ducts for elimination of superfluous spirits: I+K=through the nose, L=through the palate.

 The cerebral circulation is provided by large arteries that branch to supply the external surface, the bottom of the cavities, and the pineal gland, carrying blood formed by “its most lively, strongest, and finest part”, destined to nourish the brain matter, and to produce the “animal spirits” (*esprits aninaux*). Those are “like a very subtle wind, or rather a very pure and vivid flame”. The blood that reaches the pineal gland is filtered through its numerous very small holes that allow the passage of only the finest particles into the cavities, maintaining them filled, and flowing continuously to the tubules of the internal net and to the pores between the filaments of the external space, and beyond.^16^ ^,^ ^18^

## THE FUNCTION OF THE CARTESIAN BRAIN

The inflow of the animal spirits into the cavities and the tubules dilates the brain and makes it capable of many functions - wake up or sleep, receive impressions from external objects, imprint ideas of these qualities as common sense and imagination, retain these ideas in the memory and recall them, provide internal movements of the appetites and passions, and impart external movements of all parts of the body.^16^

Such accomplishments are due to the characteristics of the tubules and the filaments, whose main quality is their flexibility (“almost as they were made of lead or wax”), pushed by the force of spirits that touch them. Their pores allow the passage of the animal spirits, whose course is regulated by movements of the pineal. The filaments can change their shape, and the pores can be variably enlarged or narrowed accord ing to the force of the inflowing animal spirits, a mecha nism that supports the functional basis for the facul ties (attention, sensory perception, memory), and other functions of the brain.^16^ ^,^ ^18^

The pineal gland is especially important in Descartes’ project. There is lodged the main part of the soul (“rational soul”) [mind], source of all thoughts and wills (volitions, motivations, wishes), as well as the common sense and imagination. The gland centralizes most activities through its movements, directing the spirits that it releases to different parts of the brain, as necessary. The gland can be moved by the soul, by the force of the spirits that it releases, keeping it erect and immobile, or agitated and tilting to direct the spirits to tubules of different parts of the brain, and through the stimulation of the sense organs.^16^ ^,^ ^18^ It is important to remember that Descartes was preceded, on the debate about the localization of the soul, by many notable authors (Box 1).

**Box 1.** The localization of the soul before Descartes*.*

The ancient scholars felt a necessity to localize the soul in a specific organ or anatomic structure. Thus, in Egypt (3^rd^ millennium BC) the soul was seen as composed of five parts, being the most important placed in the heart, the seat and source of thoughts, feelings, and will.^25^ Much later, in Greece, philosophers, such as Socrates (5^th^-4^th^ century BC), Platon (5^th^-4^th^ century BC), and Aristoteles (4^th^ century BC) discussed the subject. Platon, based on Socrates’ ideas, considered a tripartite soul, differentially located, the *logos* (reason) in the brain, *thymos* (spiritedness) in the thorax, and *eros* (appetitive) in the stomach. Aristoteles regarded the soul linked to the body, and also constituted by three parts, a vegetative, a sensitive, and an intellectual, all related to the heart. Further, Galenus of Pergamon (3^rd^-2^nd^ century BC) adopted Platon’s idea of a tripartite soul, and located the rational soul in the brain, the spirited in the heart, and the appetitive in the liver.^25^ ^,^ ^26^ ^,^ ^27^ The Hellenistic theories of Epicurus and the Stoics that appeared, considered that the soul was corporeal.^27^ Nemesius of Emesa (4^th^ century AD), in the beginning of the Middle Ages, founded on Herophilus and Erasistratus, and also on Galenus’ ideas, believed that the soul was designed for unification with the body, and posited his ‘ventricular doctrine’, where the brain ventricles were occupied by the faculties of the soul. This view was held, with some variations, over the entire Middle Ages and the Renaissance.^4^ ^,^ ^28^ At this time appeared Descartes with his ‘mind-body dualism’, and believed that the soul has its principal localization in the brain, and specifically the pineal gland.^16^

The function of the senses is elicited by stimuli that originate from a given source (object), such as light, sound, smell, touch, heat, and other qualities, which come in contact with the [terminal] small filaments lodged in the sense organs, which in turn results in the opening of adequate small tubules in the interior surface of the brain, outlining there a figure (image), and affecting also the pineal gland. The gland releases more spirits that open further the already selected tubules, and the figure [image] related to the real object is charted in the interior surface of the brain, and on the surface of the gland [perception].^16^ ^,^ ^18^ ^,^ ^19^ The function of the sense organs, underlying perception, may further be involved in memory mechanisms, leaving tubules partially open [traces], easing memory formation; retaining images [memory], dependent of the strength, duration, and repetition of the action of the spirits, and recovering them at a later time [recollection], such activity occurring in the external region of the solid part of the brain.^16^

## COMMENTARIES

Descartes was a personage with a brilliant and creative mind, a fact that nobody can deny. He regarded the solid matter or the brain differently from the authoritative researchers known at his time, who saw it as an amorphous matter devoid of any specific function. In a different manner, Descartes proposed, for the first time, a minute structure for this solid matter, although hypothetical, and based on it, a presumptive function. More than three decades after Descartes’ description, and fifteen years after his death, the microscopic structure of the brain was revealed by Marcello Malpighi, first in letters (1665), and then in his *Viscerum Structura* (1666), with its deep structures, and an external layer, the cerebral cortex, constituted by packed small elements (neurons).^20^ ^,^ ^21^ ^,^ ^22^ ^,^ ^23^

**Figure 3.**
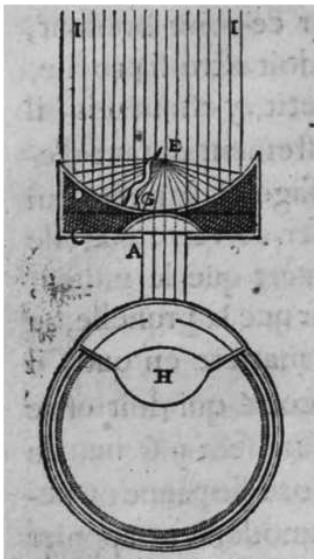
Descartes design for an instrument to amplify and visualize small objects. He described (*La Dioptrique* [9^th^ Discourse - *Des Lunettes*]) a lunette (*lunete*), an unilenticular instrument, where solar rays (I) are focused on the object (E) supported by a small arm (G), by a concave mirror (D) with a central transparent area behind which is placed the crystal (*verre*) [lens] (A), embedded in a supporting structure (C). The rays are first reflected to the object, and then, from the object to the eye (H).^19^

Interest in the minute structure of natural objects appears to have especially developed towards the end of the 16^th^ and during the beginning of the 17^th^ century. In this period, a number of scientists projected or constructed instruments to see the amplified structure of plants and animals. Among those may be cited Zacharias Jansen (1590), Galileo Galilei (1623), René Descartes (1637), Robert Hooke (1665), and Antoni van Leeuwenhoek (1673), among others.^24^ Descartes demonstrated his interest in the minute (microscopic) world, considering that among his projects of instruments to improve the vision (lunettes of varied lengths) in his *La Dioptrique* (1637), he presented a schema of an instrument intended to amplify small objects [microscope] (Figure 3).

It is unlikely that he actually used such instrument to analyze tissues, as he never mentioned such fact. However, he was aware of the potential of the ability to see the minute structure of the tissues, as he wrote (*La Dioptrique* [10^th^ Discourse]): “…And so the difficulty that you can find in the construction of the mentioned lunettes should not repulse you… I however find them much more useful because we will be able to see by their means the various mixtures and arrangements of the small parts of which are composed animals and plants…and from there get much advantage to come to the knowledge of their nature…” (1637).^6^ ^,^ ^19^ He also was used to abstract thinking, as he was an outstanding mathematician and philosopher. Thus, with this possibility in mind, he could have imagined the “fine” structure of tissues, including nervous tissues, when he posited his design. Perhaps one could speculate that it was not the result of his fantasy only, but a sort of a daring prediction.

Regarding the functional aspect, he devised the brain as a machine, with a fluid, the animal spirits, flowing inside tubes, a hydraulic device. Such way of thinking may be understood, i.e., the application of physics and its rules, one of the cherished subjects of his studies, to the mechanisms of the animal body functioning.^10^

Descartes, thus, provided, although in a hypothetical manner, the first structural and functional theories of the nervous system, the understanding of cognitive processes and of sensory and motor activities.
